# Disinhibited social engagement and reactive attachment behaviours in children assessed for Fetal Alcohol Spectrum Disorder (FASD): Distinguishing effects of maltreatment from inhibitory control difficulties

**DOI:** 10.1007/s10802-026-01446-x

**Published:** 2026-03-27

**Authors:** Ned Chandler-Mather, Erinn Hawkins, Dianne Shanley, Eva Samios, Laura Read, Sharon Dawe

**Affiliations:** 1 School of Applied Psychology, Griffith University, Level 7 Ian O’Connor Building (G40) Parklands Drive, Southport, QLD 4222 Australia; 2 School of Applied Psychology, Griffith University, Brisbane, QLD 4111 Australia

**Keywords:** Attachment disorder, Maltreatment, Neurodevelopment, Fetal alcohol spectrum disorder (FASD), Reactive attachment disorder (RAD), Disinhibited social engagement disorder (DSED)

## Abstract

The developmental origins of reactive attachment disorder (RAD) and disinhibited social engagement disorder (DSED) continue to be poorly understood. This cross-sectional study reports on the rates of RAD and DSED behaviours in a clinical sample of children presenting for Fetal Alcohol Spectrum Disorder (FASD) assessment (*N* = 123). It examines whether RAD and DSED behaviours, assessed by diagnostic interview with carers, are better explained by maltreatment (timing/type) or neurodevelopmental inhibitory-control deficits, assessed using the BRIEF-2, adjusting for age, sex and variables related to care history. There were high rates of DSED and relative low rates of RAD behaviours in this sample. Neglect past 24 months of age significantly predicted the presence of DSED behaviours related to affectionate behaviour and lack of reticence with strangers, whereas neglect and removal before 24 months did not impact these behaviours. Poor inhibitory control significantly predicted most DSED behaviours, and a RAD behaviour related to dysregulated negative affect. The overlap between poor inhibitory control, which was unrelated to maltreatment exposure in this sample, and DSED and RAD behaviours highlights the risk of diagnostic confusion when assessing for these disorders in children with suspected FASD. However, the findings also emphasise that even in the presence of poor inhibitory control owing to alcohol exposure, co-occurring exposure to prolonged neglect (past 24 months) may still contribute to the presentation of DSED behaviour. Implications for diagnosis and support are discussed. Future work should determine the non-inhibitory control related mechanisms contributing to DSED and RAD in children with prenatal adversity.

Reactive attachment Disorder (RAD) and Disinhibited Social Engagement Disorder (DSED) are both associated with early adverse caregiving experiences, particularly neglect, yet their aetiology remains incompletely understood. Other neurocognitive factors, such as poor inhibitory control, are associated with the significant emotional dysregulation and social disinhibition characteristic of these disorders, but can stem from neurodevelopmental disorder or prenatal adversity rather than postnatal adverse caregiving environments (Zeanah & Gleason, 2015). Empirical studies suggest that RAD and DSED symptom clusters are generally distinct in both children and adolescents (Gleason et al., 2011). However, controversy remains over whether RAD and DSED represent unique syndromes related to attachment or are simply different behavioural expressions of the neurodevelopmental sequalae of early deprivation (Lyons-Ruth et al., 2015; Zeanah & Gleason, 2015). High comorbidity with other conditions, such as Attention-Deficit/Hyperactivity Disorder (ADHD), Autism, and Fetal Alcohol Spectrum Disorder (FASD) can also make differentiating between conditions challenging (Clark et al., 2024; Follan et al., 2011; Minnis et al., 2013; Sarr et al., 2025). Studies have focused on disentangling RAD and DSED from ADHD (Follan et al., 2011) and Autism (Sarr et al., 2025), however, studies on FASD are lacking.

This study aims to clarify whether RAD and DSED behaviours in 3–10-year-old children prenatally exposed to alcohol presenting at a specialised FASD diagnostic clinic reflect the effects of exposure to neglect and other forms of maltreatment, or whether they represent the confounding influence of poor inhibitory control that is often associated with the teratogenic effects of prenatal alcohol exposure and FASD. The study focuses on poor inhibitory control, given evidence highlighting its aetiological role in DSED in particular *(e.g.*, Gleason et al., 2011). The timing of neglect will also be considered to identify whether a critical period for the development of either disorder exists, as has been identified in children from institutional settings (Smyke et al., 2012).

## Two Disorders with Shared Origins: RAD and DSED

Children who experience profound neglect in infancy and early childhood may go on to develop significant emotion dysregulation and abnormal patterns of relating to caregivers and strangers (Zeanah & Gleason, 2015). Two distinct patterns of abnormal relating and emotion dysregulation have been described: Reactive Attachment Disorder (RAD) and Disinhibited Social Engagement Disorder (DSED) (American Psychiatric Association, 2013; Gleason et al., 2011; Zeanah & Gleason, 2015). RAD is defined by a constellation of emotionally withdrawn, inhibited behaviour and is characterised by difficulties with seeking or accepting comfort or closeness with others, limited positive affect, and unpredictable or erratic moods (Zeanah & Gleason, 2015). The converse pattern is observed in DSED, which is defined by a constellation of indiscriminate, disinhibited social relating behaviour and is characterised by overly friendly or affectionate overtures towards unfamiliar adults and a lack of reticence in new spaces (Zeanah & Gleason, 2015). Diagnostic criteria for both disorders require that the child has been exposed to insufficient care that is disrupted and/or neglectful. Beyond the seminal findings from Romanian orphanages, where children experienced institutional neglect, RAD and DSED behaviours have also been reported in children exposed to neglect in community settings (Boris et al., 2004; Bruce et al., 2009; Minnis et al., 2013; Smyke et al., 2012). Children diagnosed with RAD or DSED were found to have significantly poorer self-esteem compared to children who experienced the same care but did not develop either disorder (Seim et al., 2021). These diagnoses are also linked to greater difficulties with social functioning later in life relative to the general population (Davidson et al., 2024), and marked comorbid internalising and externalising psychopathology (Seim et al., 2022).

## Competing Pathways to RAD and DSED: Attachment Disturbance Vs. Neurocognitive Impairment

The developmental pathways that underlie RAD and DSED are still poorly understood. Two primary explanations have been proposed: one highlights the role of neglect in disrupting foundational attachment relationships and the cascading effects on the attachment system (Lyons-Ruth et al., 2015), while the other points to the direct effects of neglect on neurocognitive abilities, such as inhibitory control(Zeanah & Gleason, 2015).

Evidence supporting the attachment account of RAD and DSED includes studies that examine children’s responses to placement in stable care and those examining the developmental timing of neglect. Placement into stable foster care is associated with reductions in RAD and DSED symptoms (Bruce et al., 2019; Humphreys et al., 2017; Smyke et al., 2012). This symptom reduction has been attributed to changes in attachment representations over time, given that placement stability promotes more secure attachment representations in young children (Steele et al., 2024). Qualities of the parent-child relationship have been shown to predict greater disinhibited social engagement behaviour in young children post adoption, namely a permissive parenting style (DePasquale et al., 2020), further emphasising the potential role of attachment-related factors.

Interestingly, in the limited studies to date, there is converging evidence to suggest a critical period for the development of DSED behaviours in children. A study examining DSED behaviours in 68 Romanian orphans who were randomly assigned to foster care found that those placed after 24 months of age exhibited inflated rates of DSED from 30 to 54 months of age compared to those placed before 24 months of age (Smyke et al., 2012). Another study found inflated rates of DSED between 7 and 24 months of age relative to children aged between 1 and 6 months and 25 months and older (Kay et al., 2016), while another found no association with timing of neglect and removal (Humphreys et al., 2017). Interestingly, a case series of three children with RAD that had onset prior to 5 years and persisted to aged 9 to 14 years had all been removed and placed into foster or other care arrangements from 2 years to 5.5 years of age (Nelson et al., 2020). Attachment theory and empirical models of attachment stability across the lifespan suggests that infancy to early toddlerhood represents a critical period for the maturation of the attachment system, finding that the stability of the attachment system increases as children develop past toddlerhood and into middle childhood and adolescence (Fraley, 2002). Thus, exposure to neglect and placement into sufficient care prior to 24 months may offer opportunity for attachment systems to adapt, whereas some attachment disturbances may be more resistant to change if placement occurs after 24 months of age.

On the other hand, there is mounting evidence that casts doubt on whether disruptions to attachment relationships play a central role in giving rise to RAD and DSED behaviours via disturbed attachment representations (Zephyr et al., 2021). A recent meta-analysis of 21 studies found small to moderate associations between attachment insecurity and disorganisation and DSED behaviours, suggesting that processes other than attachment likely contributed to the development of DSED specifically (Zephyr et al., 2021). Similarly, a direct examination of attachment representations in children with RAD found that 30% had representations consistent with a secure attachment, which suggests there is heterogeneity in the aetiology of RAD beyond attachment representations (Minnis et al., 2009).

### The Role of Inhibitory Control in RAD and DSED

An alternative developmental account has been proposed positing neurocognitive impairment as driving the emergence of RAD and DSED (Zeanah & Gleason, 2015). Exposure to early life neglect can negatively affect neurodevelopment by depriving children of expected opportunities to learn from environmental inputs that support the maturation of cognitive abilities such as inhibitory control (Nelson et al., 2007). There is increasing evidence for the role of inhibitory control, an executive function, in driving DSED behaviours (Gorter et al., 2017; Pears et al., 2010). Children with poor inhibitory control often have marked difficulties suppressing impulses (e.g., approaching strangers or being overly familiar in unfamiliar settings). The role of inhibitory control in RAD is less clear, but it could affect emotion dysregulation and unexplained outbursts.

Two previous studies have directly examined the role of inhibitory control in DSED behaviours. Both found that the number of foster care changes (Pears et al., 2010) and the time spent in institutional care (Gorter et al., 2017) predicted poorer inhibitory control, which in turn predicted greater carer-reported behaviours of DSED. In the case of children in institutional care, poorer inhibitory control has been shown to fully mediate the relationship between duration in care and DSED behaviours (Gorter et al., 2017). These findings suggest that DSED may, in some cases, reflect neurodevelopmental impairments in inhibitory control arising from environmental deprivation, rather than an attachment-related disorder.

## FASD and PAE as a Helpful Test Case

The issue of whether to attribute DSED and RAD behaviours to either attachment-related disturbance or poor inhibitory control resulting from exposure to neglect is particularly salient for children with Fetal Alcohol Spectrum Disorder (FASD), a disorder characterised by a spectrum of neurodevelopmental and physical impairment due to prenatal alcohol exposure (PAE) (Wozniak et al., 2019). In addition to neurodevelopmental impairment, children with PAE are at a heightened risk for developing an attachment disorder relative to children from the general population (Clark et al., 2024). The high rates of postnatal neglect and other forms of maltreatment experienced by children with PAE and FASD (Flannigan et al., 2021) may contribute to their inflated risk for comorbid RAD or DSED diagnosis.

The rate of RAD and DSED behaviours separately in children with FASD has not been addressed to date. There has been interest in whether certain genetic or neurodevelopmental profiles may predispose children to one form of attachment disorder over another (Zeanah & Gleason, 2015). Children with FASD present with a generally dysexecutive neurodevelopmental profile, which may predispose them to higher rates of DSED symptoms as a result of exposure to neglect.

Furthermore, whether the development of RAD and DSED behaviours is conferred through consequent impaired inhibitory control or other maltreatment related processes (e.g., attachment processes) is unclear. Another thus far unexamined possibility is that the marked neurocognitive impairments associated with FASD, especially to inhibitory control (Kingdon et al., 2016; Rasmussen, 2005), may produce overlapping behaviours with DSED and RAD, independent of maltreatment. Previous research on children with ADHD (Follan et al., 2011) and ASD have demonstrated that children with neurocognitive impairments can present with behaviours that overlap with diagnostic descriptions of RAD and DSED behaviours. Clarifying whether RAD and DSED behaviours in children with FASD reflect maltreatment-related processes, poor inhibitory control, or a combination of both will help disentangle their aetiology and reduce diagnostic confusion.

The current study aims to examine the unique contributions of exposure to neglect and other types of maltreatment and poor inhibitory control to DSED and RAD behaviours in a sample of children with prenatal alcohol exposure who have undergone assessment for FASD in specialist diagnostic clinics. Examination of the unique effects of different types of maltreatment on behaviours of DSED and RAD has been limited to date (Lehmann et al. 2020a, b). In addition to all children having prenatal alcohol exposure, which can impair inhibitory control, some of these children have experienced maltreatment and have been placed into out of home care while some have experienced stable care. Thus, this sample allows for the examination of the unique effects of poor inhibitory control, the timing of neglect, and exposure to other types of maltreatment on RAD and DSED behaviours. Although the clinical database did not include a measure of attachment behaviour or internal representations, developmental history data provided information on the timing of neglect exposure. This made it possible to distinguish whether neglect occurred during a critical period in the formation of attachment relationships, specifically before or after 24 months of age, an interval previously shown to differentially affect DSED behaviours.

If neglect exposure predicts RAD or DSED behaviours while controlling for poor inhibitory control, this would support a unique aetiological role for neglect. Should this effect be limited to neglect that extends beyond 24 months of age, this would provide evidence that disruption to the formative period of attachment relationships can lead to persistent relational disturbances later in childhood (Smyke et al., 2012). Alternatively, if poor inhibitory control alone, or both neglect and inhibitory control, significantly predict RAD and DSED behaviours, this would suggest that both factors independently or jointly contribute aetiologically. Importantly, if poor inhibitory control is unrelated with any other maltreatment exposure, this would suggest that non-maltreatment related inhibitory control impairment may present as RAD and/or DSED-like behaviours in children with FASD, highlighting phenotypic overlap similar to that detected in ADHD studies (Follan et al., 2011).

It was hypothesised that poor inhibitory control would be unrelated to maltreatment in this sample of children with PAE and FASD, given that PAE was hypothesised to be the key driver of poor inhibitory control in this sample (Kingdon et al., 2016). In line with previous research, exposure to neglect was hypothesised to have significant unique effects on all DSED behaviours (Gorter et al. 2017; Lehmann et al. 2020a, b; Pears et al. 2010) and exposure to neglect and physical and emotional abuse were hypothesised to significantly predict RAD behaviours (Lehmann et al. 2020a, b). For both DSED and RAD, it was hypothesised that exposure to neglect past 24 months of age would place children at a significantly higher risk for DSED and RAD behaviours compared to the risk for those with exposure that ended before 24 months of age, in line with a previous finding and the putative role of attachment processes that are consolidating during this period (Smyke et al., 2012). Given previous research establishing a robust link between inhibitory control and DSED behaviours (Gorter et al., 2017; Pears et al., 2010), it was hypothesised that inhibitory control, unrelated to maltreatment, would predict DSED behaviours but not RAD behaviours, suggesting symptom overlap similar to ADHD.

## Method

### Participants

Participants were children aged between 3 and 10 years referred to a specialist, multidisciplinary neurodevelopmental assessment clinic for comprehensive evaluation. Participants were eligible for inclusion in the study if: (a) their carer consented to the use of their assessment information for research; (2) there was a completed clinical interview with the carer on the chart; (3) there was a completed measure of inhibitory control on the chart; and (4) they had received a diagnosis of FASD or an ‘At Risk of FASD’ designation, as per the Australian Guide to the Diagnosis of FASD (Bower & Elliott, 2016). A total of 123 were eligible to participate, with 26 children excluded due to missing inhibitory control (*n* = 4), missing over half of their attachment interview in their chart (*n* = 18), or not having received a diagnosis of FASD or designation of “At risk” of FASD (*n* = 4).

The final sample consisted of 123 children aged between 3 and 9.5 years and their carers. See Table 1 for a description of the sample.

**Table 1:** Descriptive statistics for total sample (*N* = 123)

Variable	Outcome
Child age in months, M (SD)	71.40 (16.21)
Child sex (Male), n (%)	68 (55.3)
First Nations, n (%)	58 (47.2)
Primary carer, n (%)	
Adopted	1 (0.8)
Biological carer	31 (25.2)
Foster carer	42 (34.1)
Kinship carer	48 (39.0)
Residential care	1 (0.8)
Number of placement changes, M (SD)	1.35 (1.45)
Age removed into care in months, M (SD)	11.21 (17.60)
Age placed into current care in months, M (SD)	20.13 (23.99)
Time spent in current placement, months, M (SD)	51.09 (23.33)
Proportion of age in current placement, M (SD)	0.73 (0.31)
Number of children in the home, M (SD)	2.35 (2.02)
FASD status, n (%)	
‘At Risk’ designation	43 (35.0)
FASD < 3SFF	70 (56.9)
FASD with 3SFF	10 (8.1)
ADHD comorbidity, n (%)	71 (57.7)

FASD <3SFF refers to FASD with less than three Sentinel Facial Features and FASD with 3SFF refers to FASD with three Sentinel Facial Features

### Study Design and Procedure

This observational, cross-sectional study used retrospective chart audit data from a multidisciplinary neurodevelopmental assessment clinic for children 3 to 10 years of age suspected of prenatal alcohol exposure. Data were extracted by trained coders from clinical records of eligible participants who presented to the clinic between May 2018 and May 2024. Data included standard intake documentation, psychological assessments, and structured clinical interviews with carers. See Dawe et al. (2023) for a detailed description of the FASD diagnostic procedures at the clinic. Institutional and hospital ethical review was provided by human research ethics committees. Written consent was provided by the child’s legal guardian prior to assessment.

### Measures

#### Demographics

Demographic information, including child age, gender, number of placement changes, duration of the current placement, diagnostic status, and comorbid Attention Deficit Hyperactivity Disorder (ADHD) diagnosis was extracted from clinician documentation and intake forms available in each child’s chart.

#### Exposure to Maltreatment

Five types of exposure to maltreatment were coded for each child based on interview and collateral information in the child’s chart, including: exposure to neglect, physical abuse, emotional abuse, sexual abuse, and domestic violence (see Supplementary Table 1 for detailed description of the coding definitions). Criteria for coding each type of maltreatment were defined based on the definitions used in the ACE Questionnaire (ACE-Q), except in the case of domestic violence where damage to a pet or object was included in the definition based on criteria used in the Australian Child Maltreatment Study (ACMS) (Mathews et al., 2021). Exposure to neglect was defined based on Child Safety reports of removal from care for this reason. Timing of neglect exposure was determined based on the age at which the child was removed from the neglectful caregiving environment, and was classified into one of two categories: neglect between 0 and 24 months, indicating that the child was removed from the neglectful environment before 24 months of age, and neglect after 24 months, indicating that the child remained in the neglectful environment beyond 24 months. A subset of cases (23%) were independently reviewed by a second rater to establish inter-rater reliability. Inter-rater agreement was excellent at91.72% (kappa = 0.83), with conflicts resolved via consensus.

#### DSED and RAD Behaviours

DSED and RAD behaviours were coded from aa clinical interview with carers. Interview questions were structured around the diagnostic behaviours of RAD and DSED (DSM-V; American Psychiatric Association, 2013) and map onto validated interview protocols for attachment disorders (Lehmann et al. 2020a, b). Carer responses were coded to determine the presence or absence of each RAD and DSED sign (see Table 2).

**Table 2:** Clinical interview questions mapped onto DSM-V symptoms for RAD and DSED

RAD Diagnostic Criteria	Clinical Attachment Interview Items and Questions
A. A consistent pattern of inhibited, emotionally withdrawn behaviour toward adult caregivers, manifested by both of the following:	
1. The child rarely or minimally seeks comfort when distressed.	1) Does not seek comfort: Who does your child prefer comfort to receive from when they are hurt/upset? (Coded yes if child does not seek comfort from anyone)
2. The child rarely or minimally responds to comfort when distressed.	2) Does not respond to comfort: How does your child react when comfort is offered by you (their caregiver)/another family member (including physical contact?) (Coded yes if child does not respond positively to comfort from anyone)
B. A persistent social and emotional disturbance characterized by at least two of the following:	
1. Minimal social and emotional responsiveness to others.	5) Dismissive: Does your child prefer to avoid social interaction/child dismissive of new people? 6) Avoids eye contact: Do they avoid eye contact?
2. Limited positive affect.	3) Displays limited positive affect Does your child display/experience few positive emotions (joy/pride)?
3. Episodes of unexplained irritability, sadness, or fearfulness that are evident even during nonthreatening interactions with adult caregivers.	4) Unexplained episodes of negative affect towards carer: Does your child become very angry, fearful or sad towards their caregiver for no apparent reason? Are they unpredictable in behaviour towards caregiver?
DSED Diagnostic Criteria	Clinical Attachment Interview Items and Questions
A. A pattern of behaviour in which a child actively approaches and interacts with unfamiliar adults and exhibits at least two of the following:	
1. Reduced or absent reticence in approaching and interacting with unfamiliar adults.	1) Overly friendly with strangers: Is your child overly friendly 2) Physically affectionate with strangers Coded if there was any mention of physically affectionate behaviour with strangers.
Overly familiar verbal or physical behaviour (that is not consistent with culturally sanctioned and with age-appropriate social boundaries).	3) Asks intrusive questions with strangers: Does your child ask strangers very personal or intrusive questions or discuss overly personal information? 4) Enter areas that are out of bounds: Does child tend to enter areas or explore things that other child would know are out of bounds?
Diminished or absent checking back with adult caregiver after venturing away, even in unfamiliar settings.	5) Wanders away in unfamiliar places: Does your child wander away from caregiver when visiting unfamiliar places?
Willingness to go off with an unfamiliar adult with minimal or no hesitation.	6) Goes off with a stranger: Would your child go off with a stranger?
B. The behaviours in Criterion A are not limited to impulsivity (as in attention-deficit/hyperactivity disorder) but include socially disinhibited behaviour.	7) Needs to be the centre of attention: Does your child need to be the centre of attention?

Each sign was coded as a 1 if it was rated as present and a 0 if it was rated absent and treated as a separate binary outcome. For DSED there were seven behaviours: (1) Overly friendly with strangers, (2) Physically affectionate with strangers, (3) Asks intrusive questions with strangers, (4) Enter areas that are out of bounds, (5) Wanders away in unfamiliar places, (6) Goes off with a stranger, and (7) Needs to be the centre of attention. For RAD behaviours, there were six behaviours: (1) Does not seek comfort, (2) Does not respond to comfort, (3) Displays limited positive affect, (4) Unexplained episodes of negative affect towards carer, (5) Dismissive, and (6) Avoids eye contact.

Responses were independently reviewed by two registered psychologists with disagreements resolved by consensus. If information was missing in the chart for a given item (*n* = 50 (40.65%) were missing data, median = 1), the sign was presumed absent. However, if RAD sign A.2 was missing and RAD sign A.1 was endorsed, the response from A.1 was used to impute missing data (*n* = 20). Inter-rater reliability across all behaviours was adequate (agreement = 88.20%, kappa = 0.76). Inter-rater reliability across DSED items ranged from substantial to almost perfect (agreement = 84.73% − 94.66%, kappa = 0.69–0.89.69.89). Among RAD items, one item exhibited moderate reliability, “does not respond well to comfort”, with 74.05% agreement (kappa = 0.48). The remaining items exhibited substantial to almost perfect (agreement = 84.73% − 92.36%, kappa = 0.69 − 0.85). Individual behaviours and sign counts for each disorder were selected as outcomes to be modelled.

#### Inhibitory Control

Inhibitory control was assessed using the Behavior Rating Inventory of Executive Function – Preschool edition (BRIEF-P) Inhibit scale for children aged 2 to 5 years (*n* = 56) and the BRIEF- Second edition (BRIEF-2) Inhibit scale for children aged 5 to 18 years (*n* = 67) The BRIEF-P and BRIEF-2 questionnaire forms are 63-item questionnaires, respectively, that ask carers to rate how often everyday behaviours that are indicative of poorer executive functioning have been a problem in the last 6 months. Item scores are summed to yield *T*-scores, normed based on age and gender, for scales and more global indexes that assess different aspects of executive functioning (higher scores indicate poorer executive control). The measures demonstrate high internal consistency (alphas = 0.90–0.93.90.93), and strong test-retest reliability (*r*s = 0.85–0.91.85.91), and clear correlations with other measures of child attentional and behavioural difficulties (Duku & Vaillancourt, 2014; Gioia et al., 2003, 2015; Jacobson et al., 2020). They have also been previously used to examine inhibitory control in studies examining the effects of disinhibition on DSED behaviours (Gorter et al., 2017). The BRIEF-P and BRIEF-2 Inhibit scales contain 16 and 10 items, respectively, that tap into children’s abilities to control impulses and discontinue inappropriate behaviour in everyday settings (e.g., plays carelessly or recklessly in situations where they could get hurt, interrupts others). The BRIEF-P and BRIEF-2 inhibit scales have been shown to correlate with performance on direct tasks requiring inhibition of impulsive behaviour (Garon et al., 2016; Neale et al., 2018; Soltani et al., 2023).

### Data Analysis

All analyses were conducted using “*R Version 4.2.3*” (R Core Team, 2023). Welch two sample t-tests were used to compare outcomes by group. The presence of each DSED and RAD sign was modelled using binomial logistic regression. The total count of DSED and total count of RAD behaviours were modelled using negative binomial regressions. No neglect, female, and no abuse or DV were the reference values for the categorical variables. Significance was determined by a *p*-value < 0.05. Nagelkerke’s R^2^ and log-likelihood were used to describe model fit for the logistic and negative binomial regressions, respectively.

## Results

The rates of maltreatment are reported in Table 3. Neglect was the most common form of maltreatment (46%). The RAD and DSED behaviours are also reported in Table 3. Total count of DSED behaviours was 2.87 (2.20). The total count of RAD behaviours was 1.34 (1.55). As expected, neither exposure to neglect, abuse, nor domestic violence were associated with carer-rated poor inhibitory control in this sample of children with prenatal alcohol exposure and FASD (see supplementary Tables 2 and 3). Time spent in current placement differed significantly by neglect group, whereby children with no exposure to neglect had spent significantly longer in their current placement (*M* = 58.89 months, *SD* = 21.52) than children with up to 24 months of exposure (*M* = 46.92 months, *SD* = 23.78, *t*(71.11) = 2.56, *p* =.013) and children with exposure past 24 months (*M* = 32.34 months, *SD* = 14.88, *t*(41.98) = 6.14, *p* <.001).

**Table 3:** Description of the rate of maltreatment and RAD and DSED symptoms

Variable	Frequency, *n* (%)
*Maltreatment type*	
Neglect	57 (46.3)
Neglect ≤ 24 months	38 (30.9)
Neglect > 24 months	19 (15.4)
Physical abuse	18 (14.6)
Emotional abuse	24 (19.5)
Sexual abuse	13 (10.6)
Domestic violence	47 (38.2)
*DSED symptoms*	
1) Overly friendly with strangers	56 (45.5)
2) Physically affectionate with strangers	39 (31.7)
3) Asks intrusive questions with strangers,	31 (25.2)
4) Enter areas that are out of bounds	64 (52.0)
5) Wanders away in unfamiliar places	55 (44.7)
6) Goes off with a stranger	47 (38.2)
7) Needs to be the centre of attention	61 (49.6)
*RAD symptoms*	
1) Does not seek comfort	15 (12.2)
2) Does not respond to comfort	19 (15.4)
3) Displays limited positive affect	12 (9.8)
4) Unexplained episodes of negative affect towards carer	39 (31.7)
5) Dismissive	34 (27.6)
6) Avoids eye contact	46 (37.4)

### The Effect of Neglect, Other Types of Maltreatment, Care History, and Poor Inhibitory Control on DSED Behaviours

The results of the logistic regression models predicting each DSED sign and the negative binomial model predicting the total count of DSED behaviours are displayed in Table 4. Neglect exposure past 24 months of age (relative to no exposure to neglect) and greater BRIEF Inhibitory control index scores (meaning poorer inhibitory control) predicted significantly greater odds of exhibiting overly friendly behaviour with strangers. Greater age, being male, and having experience emotional abuse predicted significantly lower odds of this behaviour. The same pattern was exhibited for exhibiting physically affectionate behaviour with strangers. Only greater BRIEF inhibitory control index predicted greater odds of asking intrusive questions with strangers and entering areas that are out of bounds. A greater BRIEF index and greater proportion of life spent in the current placement predicted greater odds of wandering away in unfamiliar places, while greater age predicted lower odds. Neglect past 24 months of age and greater BRIEF inhibitory control index predicted significantly greater odds of going off with a stranger, while greater age predicted significantly lower odds. There were no significant predictors of needing to be the centre of attention.

**Table 4:** The impact of disinhibition, timing of neglect, other types of maltreatment on behaviours of DSED

Predictors	Behaviours of DSED							Total sign count
	Overly friendly with strangers	Physically affectionate with strangers	Asks intrusive questions with strangers	Enter areas that are out of bounds	Wanders away in unfamiliar places	Goes off with a stranger	Needs to be the centre of attention	
Intercept	0.39 [− 3.57, 4.35]	−1.96 [− 7.17, 3.24]	−7.34 [− 13.22, − 1.45]	−7.19 [− 11.79, − 2.60]	−4.10 [− 8.28, 0.08]	−5.46 [− 10.29, − 0.63]	−3.76 [− 7.72, 0.20]	−1.18 [− 2.53, 0.18]
Neglect ≤ 24 months	1.12 [− 0.05, 2.25]	1.25 [− 0.11, 2.62]	0.85 [− 0.41, 2.12]	0.05 [− 1.03, 1.13]	−0.12 [− 1.22, 0.98]	0.95 [− 0.22, 2.12]	0.07 [− 0.98, 1.12]	0.22 [− 0.11, 0.56]
Neglect > 24 months	**2.42 [0.59**,** 4.25]**	**3.49 [1.37**,** 5.62]**	1.79 [− 0.02, 3.60]	0.25 [− 1.34, 1.85]	0.38 [− 1.23, 2.00]	**1.98 [0.24**,** 3.73]**	0.54 [− 1.07, 2.15]	**0.62 [0.15**,** 1.10]**
Physical abuse	−0.01 [− 1.44, 1.42]	0.37 [− 1.29, 2.03]	0.15 [− 1.33, 1.63]	0.42 [− 0.96, 1.81]	−0.21 [− 1.57, 1.15]	0.05 [− 1.40, 1.51]	0.68 [− 0.72, 2.08]	0.04 [− 0.35, 0.44]
Sexual abuse	0.59 [− 0.92, 2.10]	0.98 [− 0.68, 2.64]	0.76 [− 0.74, 2.25]	0.71 [− 0.81, 2.23]	0.24 [− 1.25, 1.73]	1.30 [− 0.24, 2.83]	1.42 [− 0.22, 3.06]	0.37 [− 0.03, 0.77]
Emotional abuse	**−1.67 [− 3.01**,** − 0.32]**	**−2.19 [− 3.82**,** − 0.56]**	−1.23 [− 2.65, 0.21]	−0.58 [− 1.73, 0.56]	−0.60 [− 1.74, 0.55]	−0.75 [− 1.96, 0.46]	−0.27 [− 1.42, 0.89]	**−0.36 [− 0.71**,** − 0.02]**
Domestic violence	−0.13 [− 1.18, 0.93]	−0.68 [− 1.99, 0.63]	−0.30 [− 1.49, 0.89]	0.31 [− 0.67, 1.29]	0.59 [− 0.44, 1.61]	0.69 [− 0.35, 1.74]	0.27 [− 0.71, 1.25]	0.06 [− 0.25, 0.36]
Age	**−0.04 [− 0.07**,** − 0.01]**	**−0.05 [− 0.09**,** − 0.02]**	0.01 [− 0.02, 0.05]	0.01 [− 0.02, 0.03]	**−0.02 [− 0.05**,** 0.01]**	**−0.04 [− 0.06**,** − 0.01]**	0.01 [− 0.02, 0.04]	−0.01 [− 0.01, 0.00]
Gender (Male)	**−0.92 [− 1.79**,** − 0.05]**	**−1.05 [− 2.07**,** − 0.03]**	−0.64 [− 1.60, 0.32]	0.33 [− 0.49, 1.16]	−0.60 [− 1.42, 0.23]	−0.14 [− 1.02, 0.73]	−0.70 [− 1.50, 0.10]	−0.22 [− 0.47, 0.03]
BRIEF inhibitory control index	**0.05 [0.01**,** 0.08]**	**0.08 [0.03**,** 0.13]**	**0.06 [0.01**,** 0.11]**	**0.07 [0.03**,** 0.11]**	**0.05 [0.01**,** 0.08]**	**0.07 [0.02**,** 0.11]**	0.03 [− 0.01, 0.06]	**0.03 [0.02**,** 0.04]**
Placement changes	−0.12 [− 0.54, 0.30]	−0.04 [− 0.54, 0.47]	0.10 [− 0.33, 0.52]	0.09 [− 0.29, 0.46]	0.21 [− 0.17, 0.61]	0.22 [− 0.17, 0.62]	−0.03 [− 0.41, 0.35]	0.05 [− 0.06, 0.17]
Proportion of age in current placement	−0.64 [− 2.80, 1.51]	−0.90 [− 3.33, 1.53]	0.71 [− 1.69, 3.11]	1.45 [− 0.60, 3.50]	**2.46 [0.26**,** 4.66]**	2.04 [− 0.29, 4.37]	1.47 [− 0.63, 3.57]	0.62 [− 0.03, 1.27]
*Model fit*								
Nagelkerke’s Pseudo R^2^	0.30	0.43	0.24	0.20	0.20	0.28	0.17	-
Log Likelihood	-	-	-	-	-	-	-	−488.044

Table cell values include regression coefficients and 95% CIs

### The Effect of Neglect, Other Types of Maltreatment, Care History, and Poor Inhibitory Control on RAD Behaviours

The results of the logistic regression models predicting each RAD sign and the negative binomial model predicting the total count of RAD behaviours are displayed in Table 5. Exposure to physical abuse predicted significantly greater odds of not seeking comfort and not responding positively to comfort. Greater BRIEF inhibitory control index scores (poorer inhibitory control) predicted significantly greater odds of exhibiting unexplained episodes of negative affect. No other model demonstrated significant predictors.

**Table 5:** The impact of disinhibition, timing of neglect, other types of maltreatment on behaviours of RAD

Predictor	Behaviours of RAD						Total sign count
	Does not seek comfort	Does not respond to comfort	Displays limited positive affect	Unexplained episodes of negative affect towards carer	Dismissive	Avoids eye contact	
Intercept	−4.59 [−11.73, 2.55]	−4.23 [−10.40, 1.95]	−6.65 [−15.41, 2.10]	−11.18 [−17.75, −4.60]	−4.52 [−8.67, −0.38]	−4.65 [−8.76, −0.55]	−2.73 [−5.00, −0.45]
Neglect ≤ 24 months	−0.56 [−2.54, 1.41]	−0.03 [−1.64, 1.59]	1.17 [−0.92, 3.27]	0.44 [−0.79, 1.66]	0.66 [−0.49, 1.80]	0.68 [−0.39, 1.75]	0.34 [−0.25, 0.94]
Neglect > 24 months	−1.30 [−3.97, 1.36]	−0.40 [−2.78, 1.98]	1.93 [−0.78, 4.63]	0.10 [−1.61, 1.81]	0.10 [−1.75, 1.96]	1.35 [−0.23, 2.93]	0.33 [−0.09, 0.76]
Physical abuse	**2.66 [0.47**,** 4.86]**	**2.64 [0.73**,** 4.55]**	0.10 [−2.09, 2.29]	0.01 [−1.51, 1.53]	0.53 [−0.93, 1.99]	−0.04 [−1.38, 1.30]	0.49 [−0.20, 1.18]
Sexual abuse	0.26 [−1.80, 2.32]	−0.05 [−1.95, 1.85]	1.35 [−0.72, 3.42]	0.83 [−0.70, 2.36]	0.75 [−0.83, 2.34]	0.52 [−0.88, 1.93]	0.48 [−0.17, 1.14]
Emotional abuse	0.26 [−1.54, 2.05]	−0.30 [−1.90, 1.29]	0.68 [−0.89, 2.25]	0.63 [−0.59, 1.86]	0.33 [−0.95, 1.61]	−0.00 [−1.13, 1.12]	0.08 [−0.19, 0.37]
Domestic violence	−0.47 [−2.05, 1.11]	−0.59 [−2.05, 0.87]	0.37 [−1.24, 1.98]	0.48 [−0.59, 1.52]	−0.51 [−1.57, 0.55]	−0.62 [−1.61, 0.36]	−0.08 [−0.36, 0.19]
Age	0.02 [−0.02, 0.06]	−0.01 [−0.05, 0.03]	−0.02 [−0.07, 0.02]	0.01 [−0.02, 0.05]	0.01 [−0.01, 0.04]	0.00 [−0.02, 0.03]	0.00 [−0.03, 0.04]
Gender (Male)	0.99 [−0.40, 2.38]	0.27 [−0.85, 1.39]	0.65 [−0.81, 2.11]	0.46 [−0.47, 1.39]	0.88 [−0.06, 1.82]	0.83 [−0.01, 1.67]	0.40 [−0.04, 0.85]
BRIEF inhibitory control index	0.03 [−0.03, 0.09]	0.04 [−0.02, 0.10]	0.06 [−0.02, 0.14]	**0.11 [0.05**,** 0.17]**	−0.01 [−0.04, 0.03]	0.03 [−0.01, 0.07]	**0.02 [0.01**,** 0.04]**
Placement changes	−0.65 [−1.52, 0.22]	−0.16 [−0.75, 0.42]	−0.44 [−1.36, 0.48]	−0.21 [−0.69, 0.28]	0.31 [−0.09, 0.72]	0.16 [−0.21, 0.54]	−0.01 [−0.21, 0.19]
Proportion of age in current placement	−1.57 [−5.17, 2.04]	0.15 [−2.85, 3.14]	0.89 [−3.76, 5.54]	1.18 [−1.28, 3.63]	2.41 [−0.12, 4.94]	0.94 [−1.10, 2.98]	0.74 [−0.21, 1.69]
*Model fit*							
Nagelkerke’s Pseudo R^2^	0.24	0.17	0.31	0.22	0.26	0.13	-
Log likelihood	-	-	-	-	-	-	−378.701

Table cell values include regression coefficients and 95% CIs

## Discussion

Determining whether symptoms of reactive attachment disorder (RAD) and especially disinhibited social engagement disorder (DSED) reflect the impact of exposure to neglect, other types of maltreatment, or difficulties with inhibitory control is clinically and empirically challenging (Lyons-Ruth et al., 2015; Zeanah & Gleason, 2015). The current study examined these factors in children with FASD, who exhibit poor inhibitory control and experience high rates of maltreatment and comorbid attachment disorders. A key finding was that neglect that occurred past 24 months of age predicted significantly greater DSED behaviours compared to children who had not experienced neglect, while controlling for poor inhibitory control. In contrast, experiencing neglect and then removal into foster or kinship care before 24 months of age did not predict significantly elevated risk of DSED behaviours.

Exposure to neglect past 24 months uniquely predicted greater odds of exhibiting overly friendly behaviour with strangers, overly physically affectionate behaviour with strangers, and greater odds of walking off with a stranger relative to children with no exposure to neglect. Crucially, these effects held after controlling for carer-reported poor inhibitory control, as well as for other types of maltreatment, the number of placement changes, and the duration of their current placement. Thus, contrary to a recent finding that poor inhibitory control fully mediates the effect of early life neglect on disinhibited social behaviours (Gorter et al., 2017), the current findings suggest that the presence of behaviour indicative of DSED is unlikely to solely reflect general difficulties with resisting impulsive behaviour that may co-occur in children with prolonged exposure to neglect early in development. Instead, developmental processes other than poor inhibitory control alone likely play a role in DSED.

Several factors may explain this discrepancy. A lack of consideration of the developmental timing of neglect (Smyke et al., 2012) and the potential confounding of poor inhibitory control with other factors that co-occur with exposure to neglect in the previous study (Gorter et al., 2017) may explain these conflicting findings. The current study was conducted on children with FASD who had experienced neglect and other types of maltreatment in the community whereas Gorter et al. (2017) studied children who had experienced institutional privation in an orphanage, where the quality of neglect may diverge from that with a biological caregiver (Lyons-Ruth et al., 2019).

A key candidate mechanism that might explain a portion of the unique effect of neglect on DSED behaviours is the attachment system (Lyons-Ruth et al., 2015), which forms representations of self and others that shape interpersonal functioning and emotion regulation through to adolescence and adulthood (Fraley, 2002). The stability of attachment appears to increase after toddlerhood, suggesting more malleability in infancy and early toddlerhood (Pinquart et al., 2013) that might explain why exposure to neglect did not have an effect on DSED behaviours if placement into foster or kinship care occurred before 24 months in this study and a previous study (Smyke et al., 2012), and between 7 and 24 months of age in another study (Kay et al., 2016).

The first 24 months may represent a critical period, whereby the experiencing the onset of good-enough responsive care after this period may have a more blunted effect on attachment representations than when it is experienced earlier in the first 24 months. Prolonged neglect and subsequent disruption due to removal into care, may thus represent a potent attachment disturbance that may convey unavailability and unpredictability regarding significant caregivers. These associations with representations of caregivers may in turn encourage indiscriminate and over-eager seeking of attention and care from unfamiliar adult figures.

In this study neglect and removal into out of home care were conflated. Studies have shown associations between exposures to high-risk parenting contexts without removal and DSED behaviours. A recent study of infants who had not been removed suggested that suggest mothers with borderline personality disorder diagnoses were significantly more likely to have infants who exhibit disinhibited engagement with strangers at 12–18 months of age, which was partially mediated by frightening/disorienting parenting behaviour (Lyons-Ruth et al., 2019). How different dimensions and operationalisations of high-risk or potentially neglectful parenting (e.g., low emotional availability) relate to the emergence of disinhibited social behaviour without exposure to the significant stressor of placement removal is an open question. Relatedly, removal into foster or kinship care does not necessarily mark the onset of good-enough responsive care, and because the quality of the post-removal environment was not assessed in this study, any evidence that weighs towards a critical period should be treated with caution until additional evidence becomes available.

This study found that poorer carer-reported inhibitory control predicted significantly greater DSED behaviour overall and most individual behaviours associated with DSED in children with or at risk of FASD. Crucially, because poor inhibitory control was unrelated to neglect or other types of maltreatment in this sample of children with FASD, this suggests that poor inhibitory control in children because of their FASD may present as behaviours suggestive of DSED. This finding that non-maltreatment related poor inhibitory control is of clinical concern given that DSED is specified to result from exposure to insufficient care environments (American Psychiatric Association, 2013). Findings from the current study in children with FASD and a previous study in children with ADHD (Follan et al., 2011) both indicate that behaviours of DSED that related to interactions with others (e.g., physically affectionate behaviour rather than venturing into unfamiliar spaces) appear to be most sensitive to exposure to neglect in the context of co-occurring neurodevelopmental impairments related to inhibitory control. This potential for diagnostic confusion might contribute to the heightened risk for attachment disorder diagnoses in children with FASD (Clark et al., 2024), although it is equally likely that their high rates of exposure to postnatal adversity (Flannigan et al., 2021) combined with neurodevelopmental vulnerabilities (Dawe et al., 2023) are driving this.

Exposure to emotional abuse predicted significantly less DSED behaviours in the current sample. A history of negative interactions with adult caregivers associated with emotional abuse may deter children from engaging in overly friendly or familiar ways with strangers and unfamiliar environments. Physical and sexual abuse did not uniquely predict less DSED behaviour. It is possible that the affective and self-focused nature of emotional abuse (e.g., putting down, humiliating) undermines a potential motivation for engaging in behaviours indicative of DSED, that is receiving kindness from others (Bennett et al., 2009). The relatively small number of children who had experienced sexual abuse in the current sample may explain why it did not exhibit a similar pattern.

Overall, this finding implies that maltreatment should not be considered unitary in studies of DSED and RAD because different types may uniquely affect attachment disorder presentations in divergent ways (Chandler-Mather et al., 2023), in line with the developmental principle of “multifinality” (Cicchetti & Rogosch, 1996) and should instead be decomposed into its constituent types to best understand how it influences attachment disorder behaviours (Lehmann et al. 2020a, b). Likewise, different developmental precursors, in this case PAE, poor inhibitory control, and exposure to prolonged neglect and other forms of maltreatment, can co-precipitate the same developmental outcome, DSED and RAD behaviours, in line with the principle of “equifinality” (Cicchetti & Rogosch, 1996).

Unexpectedly, neither neglect nor any other type of maltreatment predicted the overall count of RAD behaviours in this sample. This null finding appears to contradict considerable evidence linking exposure to neglect and abuse to RAD behaviours (Lehmann et al. 2020a, b; Smyke et al. 2012; Zeanah and Gleason 2015). Given that children who had experienced neglect past 24 months of age in this study had still been in their current placement for approximately 32 months, and that behaviours of RAD can largely remit by approximately 8 months post placement (Smyke et al., 2012), most RAD behaviours associated with neglect and maltreatment may have remitted leading to less RAD behaviours in the current sample. Alternatively, children with FASD may be more likely to develop an externalising response to neglect in the form of DSED rather than an internalising response in the form of RAD, owing to their generally disinhibited neurodevelopmental profile and as such FASD or heavy prenatal alcohol exposure may present as predisposing factor for DSED (Kingdon et al., 2016; Zeanah & Gleason, 2015).

Exposure to physical abuse predicted significantly greater odds of carers endorsing their child not seeking comfort or responding positively to comfort compared to children without exposure to physical abuse. Physical abuse from previous caregivers may have been associated with positive punishment for care seeking behaviours leading to an avoidance of these behaviours with future caregivers. Whether physical abuse meets aetiological criteria in the DSM-5 is not necessarily clear given its emphasis on an absence of social and emotional inputs from carers rather than a commission of threatening behaviour from carers (American Psychiatric Association, 2013).

### Limitations and Future Directions

The current study featured several key strengths including the inclusion of a high-risk sample of children who experienced both pre and postnatal adversity and engagement in the child protection system, rigorous assessment of prenatal alcohol exposure and diagnosis of FASD, and the use of interview-based methods conducted by a provisional or generally registered psychologist to assess behaviours of DSED and RAD. This was the first study to explicitly control for prenatal alcohol exposure when assessing attachment disorder symptoms. However, there were some methodological limitations that provide a map for future studies to address and build on regarding assessment of DSED and maltreatment exposure.Firstly, the study focused on children diagnosed with FASD or at risk for FASD, and may have limited generalizability to other populations, like those without disorders that feature executive dysfunction. The current study used a novel clinical interview with carers based on DSM-V DSED and RAD criteria and aligns closely with established interview procedures (Lehmann et al. 2020a, b). There is currently no gold standard interview procedure for assessing RAD and DSED behaviours, however, various methods based on non-diagnostic sets of behaviours, or on DSM-IV and DSM-V nosology have been developed (Lehmann et al. 2020a, b). Use of an existing measure may have provided additional sensitivity. Observational assessments may have also provided greater fidelity regarding DSED behaviours (McMorran-Young et al., 2021).

On a related note, the current study examined behaviours of DSED rather than formal clinical diagnosis. While the diagnostic criteria for DSED relates directly to the number of behaviours endorsed by the clinician, other clinical decision making processes are involved before a formal diagnosis is made. The examination of symptoms and a count of symptoms aligns well with the approach adopted by the developmental literature on attachment disorders (Guyon-Harris et al., 2019; Humphreys et al., 2017) and with dimensional models of psychopathology (Eaton et al., 2023). However, this approach does limit the implications of these findings for formal diagnosis. Future studies could address this by adopting a consistent, formal, diagnostic approach using a combination of standardised clinical interview methodologies *(e.g.*, Lehmann et al. 2020a, b), observational tools *(e.*g., McMorran-Young et al., 2021), and clinical judgement.

In addition, more granular information regarding the timing of neglect would have allowed for a more nuanced examination regarding the pattern of exposure. Unfortunately, given that this study was based on archival clinical data that did not consistently record this level of detail or have it available from collateral reports. Future studies should attempt to leverage a larger cohort of children, more granular, dimensional measures of adversity (Berman et al., 2022), and examine patterns of co-occurring maltreatment. A larger sample would also power the study to detect smaller effects and facilitate isolating the developmental timing of adversity with more fidelity to assess whether cut-offs (e.g., 24 months used in this and prior studies: Humphreys et al., 2017; Kay et al., 2016; Smyke et al., 2012) is appropriate.

Overall, the confidence intervals for the estimated effects of maltreatment were very wide, indicating the true effect may range in size from small to very large. Thus, there remains substantial uncertainty regarding the magnitude of the impacts of different types of maltreatment on disinhibited social engagement and reactive attachment behaviours in the context of prenatal alcohol exposure. Adopting these recommendations for future studies, including implementing more sensitive measures of maltreatment exposure, and recruiting a larger sample, will provide more reliable estimates for clinicians to weigh evidence more effectively.

Future studies should attempt to identify psychological processes underpinning the unique effects of neglect and abuse on DSED and RAD behaviours (Lyons-Ruth et al., 2015; Zeanah & Gleason, 2015). Evidence converges on additional mechanisms beyond poor inhibitory control and attachment security classification. Exploration of other aspects of the attachment system, or social reward system more broadly, may help uncover additional mechanisms. Future studies should also attempt to leverage direct assessments of inhibitory control and other executive functions to provide a more comprehensive assessment of the impact of neurodevelopment impairment on DSED.

## Conclusions

Teasing apart the roles of exposure to neglect, other forms of maltreatment, and poor inhibitory control in the development of RAD and DSED behaviours is complex. The additional question of whether any contribution of poor inhibitory control represents the effects of exposure to neglect or maltreatment or whether it stems from a co-occuring neurodevelopmental disorder originating in utero adds to this complex clinical picture and has implications for diagnosis and treatment planning.

The current study unpicked the unique effects of neglect and other types of maltreatment from poor inhibitory control on DSED and RAD behaviours in young children with FASD. Neglect past 24 months of age predicted significantly greater DSED behaviours compared to children who had not experienced neglect. Physical abuse predicted significantly greater odds that a child would be rated as not seeking or responding to comfort when distressed. Poor inhibitory control, unrelated to maltreatment in this sample of children with FASD, was a significant predictor of most DSED behaviours and greater RAD behaviours, suggesting some potential for diagnostic confusion between behaviours stemming from maltreatment and those from neurodevelopmental impairment related to FASD. Disinhibited and reactive attachment behaviours in children presenting for FASD assessment requires careful assessment and formulation to disentangle disrupted attachment histories from comorbid neurodevelopmental impairments.

## Data Availability

Clinic data cannot be made publicly available due to ethical approval constraints. Analysis scripts are available upon request.
